# Effects of Intra-uterine Ceftiofur on the Equine Uterine Microbiome

**DOI:** 10.3390/vetsci12090837

**Published:** 2025-08-30

**Authors:** Kalie F. Beckers, Chin-Chi Liu, Viviane C. L. Gomes, Christopher J. Schulz, Gary W. Childers, Carleigh E. Fedorka, Jenny L. Sones

**Affiliations:** 1 Veterinary Clinical Sciences, School of Veterinary Medicine, Louisiana State University, Baton Rouge, LA 70803, USA; kbeckers@tulane.edu (K.F.B.); cliu@lsu.edu (C.-C.L.); 2 Department of Small Animal Clinical Sciences, College of Veterinary Medicine, Michigan State University, East Lansing, MI 48824, USA; leitegom@msu.edu; 3 Department of Biological Sciences, Southeastern Louisiana, Hammond, LA 70402, USA; cschulz@gulfcoastmicro.com (C.J.S.); gary.childers@selu.edu (G.W.C.); 4 Animal Sciences, College of Agricultural Sciences, Colorado State University, Fort Collins, CO 80521, USA; carleigh.fedorka@colostate.edu; 5 Clinical Sciences, College of Veterinary Medicine and Biomedical Sciences, Colorado State University, Fort Collins, CO 80523, USA

**Keywords:** equine, pregnancy microbiome, antibiotics

## Abstract

Antimicrobial therapy is a mainstay for treating reproductive diseases, including endometritis. Ceftiofur, a third-generation cephalosporin, is a common antibiotic used to treat equine bacterial endometritis. It is also routinely given empirically as an intra-uterine (IU) infusion in broodmare practice. We hypothesized that ceftiofur IU would disrupt the resident microbial community within the healthy uterus of mares. To test our hypothesis, eight university-owned mares were selected for characterization of the estrual uterine microbiome before and after IU ceftiofur. Double-guarded swabs of the estrual endometrium were taken before and 3 days after both IU saline and ceftiofur in a crossover design. Isolation of DNA from endometrial swabs was performed, followed by amplification of the V4 region of the 16S rRNA gene by Illumina Miseq sequencing to examine core bacterial communities present before and after ceftiofur. The uterine microbial composition of sham and ceftiofur-treated mares was not significantly different as measured by beta diversity. The only notable difference was a lower abundance of Christensenellaceae_R-7_group after ceftiofur (0.14 ± 1.05% vs. 2.89 ± 1.07% control; *p* = 0.0428). In conclusion, three-day treatment of ceftiofur did not change the microbial composition acutely within the mare uterus when sampled directly after treatment. Ceftiofur may have a long-term effect on the uterine microbiome, which may require sampling several weeks post treatment. In conclusion, ceftiofur does not change the healthy uterine microbiome acutely during estrus and but should still be used judiciously.

## Simple Summary

Intra-uterine infections in mares are a common cause of infertility. When they are caused by bacterial contamination of the uterus, veterinarians can treat mares with systemic or local antibiotics. Generally, local intra-uterine infusion of antibiotics that specifically target the isolated bacteria is performed to resolve infection. To prevent bacterial endometritis, empirical therapy with intra-uterine antimicrobials is commonly performed. However, it is unknown what this does to the healthy uterine microbiome of mares. Our study demonstrated that intra-uterine infusion of ceftiofur had minimal impact on the local microbiome of the mare’s uterus when she was free of signs of endometritis.

## 1. Introduction

For more than a century, the uterus has been perceived as a sterile organ. This was challenged through multiple studies utilizing uterine aerobic culture, which was determined to be free of bacteria. More recently, and due to advancements in laboratory methods (specifically 16s rRNA sequencing and whole genome sequencing), the equine uterus has been found to have resident bacteria and fungi–deemed the microbiome [1,2,3]. The reproductive tract microbiome maintains a symbiosis by suppressing invasion of microorganisms from the outside and regulating the excessive proliferation of microorganisms that are currently occupying it. When the microbial communities collapse or become unbalanced due to a variety of causes, it creates a dysbiosis, which can lead to foreign invaders or overgrowth of pathogenic bacteria, ultimately generating an inhospitable uterus [1,2,3,4,5]. However, little is known about uterine dysbiosis in the mare.

The healthy estrual mare has a core uterine microbiome that has been described and found to be similar whether using double-guarded endometrial swabs, biopsy, or low-volume lavage (LVL) [3]. However, LVL was more sensitive in detecting low taxa, but this may be dependent on geographical region [2]. Using LVL to sample the uterus of cycling mares, there is agreement that the core uterine microbiome in mares is represented by members of the Proteobacteria and Firmicutes phyla [2,3]. Characterizing the equine uterine microbiome in health compared to disease is important.

Prolonged inflammation within the endometrium, or endometritis, is a primary cause of reduced fertility in the mare [6,7]. This can be due to bacterial and fungal pathogens, or even introduction of spermatozoa during breeding [8]. Importantly, 25–60% of the broodmare population is diagnosed with bacterial endometritis [4]. Virendra et al. (2024) investigated the uterine microbiome of mares that had confirmed bacterial endometritis (n = 15; “diseased”) compared to mares with a healthy uterus (n = 15) in a single location [4]. It was found that the uterine microbiome of the diseased was vastly different from that of the healthy group. In healthy mares, the most abundant phylum, class, order, and family included Firmicutes, Bacilli, Bacillales, and Paenibacillacaea, respectively. In contrast, the most abundant phylum, class, order, and family in the diseased mares were Proteobacteria, Gammaproteobacteria, Enterobacterales, and Enterobacteriaceae, respectively. At the genus level, the most abundant bacteria in the healthy mares were *Brevibacillus* and *Paenibacillus*, while the most abundant bacteria in diseased mares were *Escherichia*, *Salmonella*, and *Klebsiella*. Therefore, mares experiencing bacterial endometritis experienced dysbiosis of the uterine microbiome. However, longitudinal assessment of the uterine milieu that allows for pathogenic bacteria to thrive is needed.

Mares susceptible to post-breeding induced endometritis (PBIE) are predisposed to chronic uterine infections [8,9] and increased early embryonic loss [10]. Due to this, treatment for PBIE commonly includes antimicrobials, such as third-generation cephalosporins (e.g., ceftiofur), beta-lactams (e.g., ampicillin and penicillin), and aminoglycosides (e.g., gentamicin and amikacin) [11]. It is unknown if antimicrobial treatment alters the uterine microbiome. Therefore, we aim to compare the uterine microbiome of the healthy estrual mares to the uterine microbiome following intra-uterine antibiotic therapy (ceftiofur) using metagenetic sequencing. We hypothesize that infusion of ceftiofur directly to the uterus of estrual mares will disrupt the resident microbial community within the uterus compared to sham infusion during estrus.

## 2. Materials and Methods

### 2.1. Animal Work

All the animal procedures were completed in accordance with the Institutional Animal Care and Use (IACUC) of Louisiana State University (LSU) under the guidelines of the approved protocol #19-079. The university-owned horses (*Equus caballus*) used in this study (n = 8) were mixed-breed mares (7–20 years of age) ranging from 450 to 550 kg housed on the same LSU pasture with hay ad libitum. Reproductive tracts were examined by a board-certified theriogenologist, and no abnormalities of the ovaries, uterus, cervix, or vagina were noted prior to the start of the study. All the animal work was completed during the 2020 summer months of the Northern Hemisphere in Louisiana, USA, with treatments being performed over the course of 2 estrous cycles.

#### 2.1.1. Sample Collection

The mares were monitored over three estrual periods (E1, E2, and E3) in a crossover design. Exclusion criteria were any mare failing to enter estrus. In the first estrous cycle, reproductive status of all the mares was followed via palpation and trans-rectal ultrasonography. For all endometrial sampling and uterine infusions, the vulva and perineum were scrubbed with 0.5% chlorhexidine three times and rinsed with tap water as routinely performed. Once the mares were determined to be in estrus (>30 mm follicle, increasing uterine edema, relaxed cervix), an endometrial culture and cytology (MOFA Global, Verona, WI, USA) were collected transcervically using a double-guarded approach to assess mares for inflammation (E1 d0). Immediately after sampling, endometrial swabs (2) were used for aerobic bacteriological culture and snap frozen for genomic DNA isolation. Sham inoculation of sterile saline was infused intra-uterine for three consecutive days (E1 d1–d3). An insemination pipette was manually placed through the cervix, and 10 mL of sterile saline was infused into the body of the uterus. On the day following the final day of saline infusion (E1 d4), the endometrium was sampled using a double-guarded swab as described above. In the next estrus, endometrial swabs were again collected similarly for culture and snap frozen for genomic DNA isolation. Following this, 1 g of ceftiofur (Naxcel; Zoetis Animal Health, Parsippany, NJ, USA), reconstituted in 10 mLs of sterile water, was infused intra-uterine for three consecutive days (E2 d1–3). An insemination pipette was manually placed through the cervix, and ceftiofur solution was infused into the body of the uterus. On the day following treatment (E2 d4), the endometrium was sampled with a double-guarded swab for genomic DNA isolation.

#### 2.1.2. Artificial Insemination

To determine the impact of dysbiosis on fertility, all eight mares were bred in the estrus following ceftiofur treatment (E3). The mares were examined daily via transrectal palpation and ultrasonography of their reproductive tracts for follicular development, endometrial edema, and uterine and cervical tone. When the presence of a pre-ovulatory follicle was noted (>35 mm) combined with reduced uterine tone, increased endometrial edema, and a relaxed cervix, the mares were inseminated and received 1500 international units of human chorionic gonadotropin (hCG; Intervet International B.V., Boxmeer, Holland, The Netherlands) intravenously to standardize the interval between insemination and ovulation. Semen was collected from a single stallion using a Missouri model artificial vagina (Nasco, Fort Atkinson, WI, USA) equipped with a gel filter (Animal Reproduction Systems, Chino, CA, USA). Only the samples with >50% progressively motile sperm at the time of collection were utilized. The semen samples were adjusted to a concentration of 500 × 10^6^ spermatozoa in 30 mL semen extender (INRA) and kept at room temperature (23–25 °C) for approximately 15 min prior to insemination. Pregnancy was assessed via ultrasonography at 14 days following ovulation.

### 2.2. Laboratory Analysis

#### 2.2.1. Endometrial Culture and Cytology

Immediately after sampling, endometrial swabs were streaked on a blood agar (5% horse blood) and MacConkey agar and incubated aerobically for 24 h at 37 °C. Bacterial growth was identified according to colony morphology and counted and scored: no growth/sterile: <5 CFU (colony forming units); mild growth: 5 to 10 CFU; moderate growth: 11 to 50 CFU; and heavy growth: >50 CFU. If growth was observed, the colonies were submitted to the LSU Disease and Diagnostic Laboratory for aerobic culture. The culture results were recorded as *Escherichia coli*, beta-hemolytic *Streptococcus* sp., and other uterine pathogens, or no growth. For endometrial cytology, brushes were smeared on glass slides, which were dried at room temperature and stained with Diff-Quik and evaluated by light microscopy (400 magnification). Cytologic classification was based on the number of polymorphonuclear neutrophils (PMNs) present per 100 endometrial epithelial cells examined [12].

#### 2.2.2. DNA Extraction, Sequencing, and Metagenomic Analyses

Genomic DNA was extracted from endometrial swabs using Qiagen DNeasy PowerSoil extraction kits (Qiagen, Hilden, Germany) as previously described [3]. In brief, DNA was extracted by removing the swab tips from the applicators with sterile razor blades and then transferring the swab material directly to bead-beating tubes. Subsequent steps followed the manufacturer’s instructions. In addition to the uterine samples, a set of blanks was processed similarly, and a negative control (no sample material was added to the bead-beating tubes). DNA extracts were visualized with gel electrophoresis, transferred to 96-well plates, and then shipped overnight on dry ice to the Research Technology Support Facility of Michigan State University for 16S rRNA sequencing using primers 515f and 806r (V4–V5 region). Barcoding and library preparation were performed, and sequencing was carried out on a Miseq platform (Illumina, Inc., San Diego, CA, USA) with 2 × 250 bp paired-end according to published protocols [3]. All the samples were sequenced twice.

The samples were filtered and trimmed based on their quality scores and error rates using the dada2 pipeline. Next, an amplicon sequence variant (ASV) table was made, and chimeras were removed. The 16S rRNA SILVA v138.1 database was used for mapping and assigning taxonomy. Next, contaminating reads were removed from the samples using Microdecon based on the negative controls (blank and no template control). Downstream analysis was performed using the Phyloseq package, version 1.44.0. Alpha diversity calculation (Shannon, Chao1, and inverse Simpson), beta diversity (weighted UniFrac), and analysis of similarity (ANOSIM statistic) were performed using the microbiome, amplicon, microeco, and vegan packages. Graphs were generated using ggplot2, dplyr, RColorBrewer, ggpubr, and lattice packages in R version 4.3.4. Bar, and pie plots were generated using Microsoft Excel. Sequences have been deposited in the NCBI SRA, PRJNA1285510.

## 3. Results

### Microbiome

None of the endometrial swabs from the estrual mares yielded bacterial growth using aerobic culture, and endometrial cytology revealed no PMNs. Next, 16S sequencing was performed on all the samples, with a minimum of 40,000 reads and up to 50 ASVs per sample. During quality control, one sample (EQ_680_UT_2) was identified as an outlier based on diversity indices and taxonomic composition (Shannon = 0.23; Kiritimatiellaeota = 95.6%) and was excluded from further analyses.

**Figure 1:**
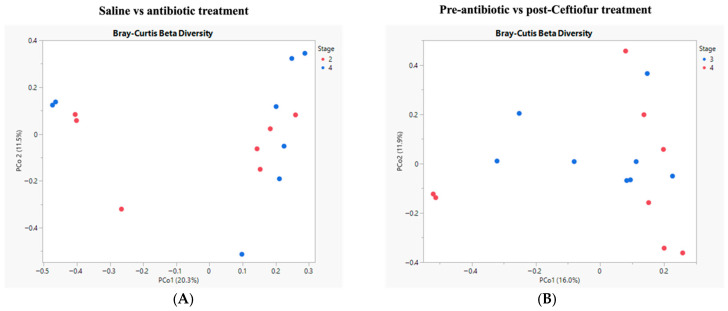
Diversity of microbial populations. The microbial community composition of sham and ceftiofur-treated mares (**A**) or pre- and post-ceftiofur-treated mares (**B**) was not significantly different. As assessed by PERMANOVA with Bray–Curtis dissimilarity of 16S amplicon sequence variants’ relative abundance.

Microbial communities were compared between pre-sham and post-ceftiofur (Figure 1A) in addition to pre-ceftiofur and post-ceftiofur treatments (Figure 1B). When assessing the microbial community composition of sham and ceftiofur-treated mares, no significant differences were noted (*p* = 0.63, PERMANOVA with Bray–Curtis dissimilarity of 16S amplicon sequence variants’ relative abundance). When assessing the microbial community composition of pre- and post-ceftiofur treated mares, no significant differences were noted (*p* = 0.32, PERMANOVA with Bray–Curtis dissimilarity of 16S amplicon sequence variants’ relative abundance).

**Figure 2:**
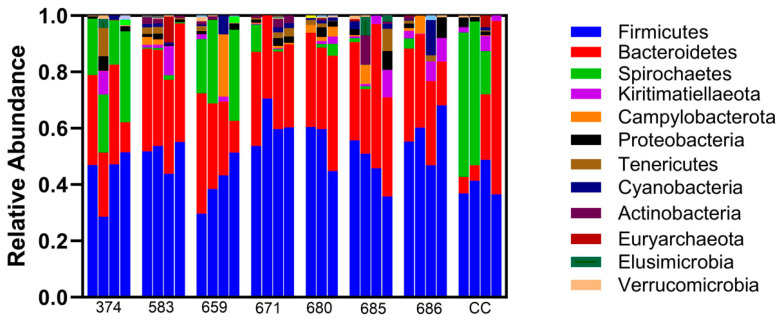
Relative abundance of bacterial DNA at the phylum level was not different between the endometria from the treatment and sham controls. Each mare is represented on the X axis, with pre-saline, post-saline, pre-ceftiofur, and post-ceftiofur represented as bars. Firmicutes, Bacteroidetes, and Spirochaetes are the most abundant phyla noted in all samples, and this did not differ based on treatment or cycle.

**Figure 3:**
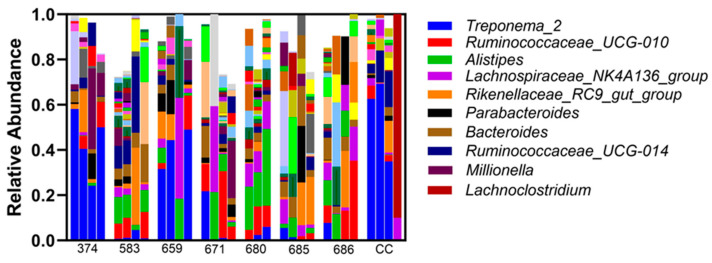
Relative abundance of bacterial DNA at the genus level was not significantly different between the endometria from the treatment and sham controls. Each mare is represented on the X axis, with pre-saline, post-saline, pre-ceftiofur, and post-ceftiofur shown. Immense variability in bacterial genus is noted across mare, but no impact of saline infusion or ceftiofur treatment was noted.

Sequence analysis of phyla identified consistent bacterial microorganisms across all the samples taken (Figure 2). Relative abundance at the phylum level was not different between the treatment and sham controls. Firmicutes, Bacteroidetes, and Spirochaetes are the most abundant phyla noted in all the samples, and this did not differ based on infusion (Figure 2). Overall, different bacterial genera were identified in endometrial samples, with Treponema_2 being of the highest abundance (Figure 3). Additionally, bacterial genus abundance was not altered in the endometrial samples compared to pre-ceftiofur to post-ceftiofur treatment.

**Figure 4:**
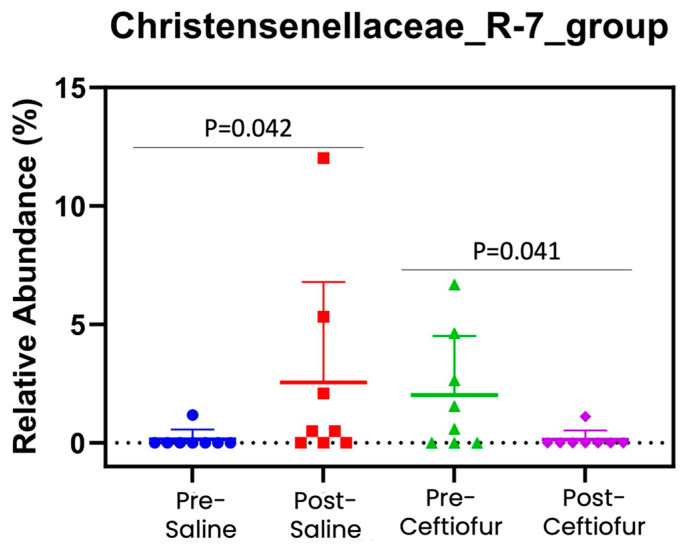
Relative abundance of Christensenellaceae_R-7_group. The only notable difference was that abundance of Christensenellaceae_R-7_group was significantly increased with saline, while reduced after ceftiofur treatment (*p* < 0.05).

Of all the bacteria evaluated, the only family that differed following sham or ceftiofur infusion was Christensenellaceae_R-7_group (Figure 4). When assessing abundance of Christensenellaceae_R-7_group between the pre-sham and post-sham samples, this family was significantly increased in the post-sham samples (0.14 ± 1.05% vs. 3.12 ± 1.07%; *p* = 0.04). In contrast, abundance of Christensenellaceae_R-7_group significantly decreased in the post-ceftiofur samples when compared to the pre-ceftiofur samples (0.14 ± 1.05% vs. 2.89 ± 1.07%; *p* = 0.04). Finally, all eight mares achieved pregnancy in the third estrous cycle as detected by transrectal ultrasonography 14 days post-ovulation.

## 4. Discussion

The equine uterus has a distinct microbiome with abundant commensal bacteria [3]. Its function is unknown, but it may provide a barrier mechanism for immunity to deter the growth of pathogenic bacteria. The use of intra-uterine antibiotic infusion for the treatment of bacterial endometritis is a common modality in equine reproduction, especially in PBIE-susceptible mares. Here, we found intra-uterine infusion of a broad-spectrum third-generation cephalosporin (ceftiofur) did not disturb the resident endometrial microbiome of estrual mares that presented with a healthy uterus. To our knowledge, this is the first study to evaluate the impact of intra-uterine ceftiofur on the uterine microbiome in the horse.

Research on the microbiota of the mare reproductive tract is a developing area of study; recent studies have indicated an influence of estrous cycle stage [3,5], geographic location [13], and disease [4]. This was specifically noted in endometritis; Virendra et al. (2024) found the most abundant phylum of the healthy estrual uterus to include Firmicutes [4]. In contrast, the most abundant phylum in the uterus of diseased mares was Proteobacteria. In the present study, the most abundant phyla mimicked that which was noted by Virenda et al. in the healthy uterus, and included Firmicutes, Bacteroidetes, and Spirochaetes. This is not surprising as our mares were clinically healthy and fertile with negative culture and cytology noted before infusion of either sham or ceftiofur. Furthermore, a 100% pregnancy rate was observed in the third estrous cycle after sham and ceftiofur-treated estrous cycles. Establishing pregnancy rates after sham treatment cycles would have aided in our knowledge of fertility and intra-uterine treatments; however, introducing semen between sham and treatment may have altered the mare’s uterine microbiome and confounded our findings.

Ceftiofur is a broad-spectrum third-generation cephalosporin and has been found effective against both anaerobic and aerobic organisms, including both Gram-positive and Gram-negative bacteria [14]. This product is commonly administered both systemically and intra-uterine, with adequate minimum inhibitory concentrations (MICs) detected in the equine endometrium [15]. In other species, ceftiofur is a potent disruptor of microbiome homeostasis, including within the gastrointestinal tract [16,17], nasal cavity [18], feces [19], and uterus [20,21]. In the present study, ceftiofur did not alter the uterine microbiome of mares, which contrasts with what has been noted in porcine and bovine models [20,21]. In the pig, intramuscular ceftiofur administration was found to alter the fecal microbiome of healthy animals, and this was specifically noted by an increase in *Prevotella*, *Bacteroides* and *Faecalibacterium* and a decrease in *Escherichia* and *Clostridium* after ceftiofur administration. The majority of studies on ceftiofur in the bovine model focused on its use for the treatment of mastitis, and therefore, ceftiofur returned the dysbiotic uterus to homeostasis. In our dataset, lack of uterine disease prior to infusion in mares may be the reason for the absence of significant community change within the endometrium after ceftiofur infusion. This is a limitation of the present study, as no aerobic bacteria were cultivated, and therefore the function of ceftiofur infusion is uncertain.

In other species, intra-uterine infusion of ceftiofur has been associated with reduced bacterial load [22] and improved fertility [23], but no studies have investigated the impact of intra-uterine ceftiofur on the microbiome. It is not surprising that infectious agents are reduced by ceftiofur upon intra-uterine administration [24], while disruption of the estrual microbiome has not been described. The normal estrual uterus will be naturally stimulated by the deposition of foreign antigens, leading to an increase in myometrial contractility and elimination of foreign particles [7,25]. This may include the ceftiofur infusion itself. This may contribute to a preference for systemic administration of antibiotics to treat uterine infection over intra-uterine infusion [26,27]. It is unknown if systemic administration of ceftiofur would have altered the uterine microbiome to a greater extent than intra-uterine infusion, and future research on this topic is deserved.

An additional finding of this study was the immense variability in abundance of bacterial genus before in addition to following intra-uterine infusion. Individual variability within the microbiome has been noted in many studies on this topic, the cause of which is often unexplained [28]. This has been mostly studied in the microbiome of the gastrointestinal tract, where diet is considered the primary cause of individual variability [29]. Multiple other aspects of health have been noted to contribute to the microbiome, including age, sex, obesity, pathophysiological status, physical activity, and ethnicity (or in the case of animals, breed) [30]. Most of these attributes were controlled for within the present study, including sex, breed, and nutrition, while obesity, physical activity, and pathophysiological status were not considered. The mares within the present study may have been experiencing underlying conditions such as osteoarthritis [31], gastrointestinal abnormalities [32], or endocrine dysfunction [33,34], all of which may alter systemic immunity and therefore the microbiome in diverse systems. Unfortunately, this was not controlled for and remains a limitation of the study at hand. Another limitation of the present study is the relatively low sample size of eight research mares. Previous studies have shown that even five mares are enough to obtain statistical significance when comparing the maternal microbiome of a pregnant horse [35]. However, a larger cohort with all confounding variables accounted for may reveal statistically significant changes in the microbiome of mares treated with intra-uterine therapy. More studies are warranted in mares with clinically diagnosed intra-uterine disease, particularly bacterial endometritis.

The introduction of Christensenellaceae_R-7_group into the mare’s uterus after sham treatment is likely a fecal contaminant. However, it has been detected in the bovine pregnant uterus [36]. In our dataset, similar relative abundance of Christensenellaceae_R-7_group was detected in the mares at the beginning of estrus following a sham cycle (E2 d1), and ceftiofur eliminated it. Iatrogenic introduction of bacteria is always a possibility with intra-uterine procedures, and thus empirical ceftiofur administration could be purposeful in eliminating low-abundance contaminants. However, antimicrobial stewardship mandates only treating an active infection, and further research is needed to determine the clinical significance of bacterial DNA in the equine uterus.

## 5. Conclusions

In this study evaluating the effect of intra-uterine ceftiofur infusion in eight estrual mares, several conclusions were made: (1) Ceftiofur did not significantly alter the uterine microbiome when the equine uterus was free of infection. (2) Iatrogenic introduction of the anaerobe Christensenellaceae_R-7_group was eliminated from the equine uterine microbiome by ceftiofur. (3) Fertility was not negatively impacted by sham nor intra-uterine ceftiofur in previous estrous cycles. Antibiotic administration is used to resolve dysbiosis, while the present study administered ceftiofur in a healthy uterine environment. This may explain the lack of dysbiosis following intra-uterine administration of ceftiofur, but this could not be confirmed within the confines of the present study. Future research is warranted using a larger cohort of clinical cases of mares with bacterial endometritis to further investigate the impact of intra-uterine administration of antibiotics on the resident bacteria in the diseased mare.

## Data Availability

No new data were created or analyzed in this study. Data sharing is not applicable to this article.
